# Supplementary material for: Impact of Spa Therapy on Symptoms and Quality of Life in Post-COVID-19 Patients with Chronic Conditions
Source: J Clin Med. 2024 Aug 27;13(17):5091. doi: 10.3390/jcm13175091 (PMC11396595; doi:10.3390/jcm13175091)
Supplement: Supplementary file 1 [file jcm-13-05091-s001.zip › jcm-3103759-supplementary.pdf]

Supplementary Materials

Supplementary Table S1. Patients' characteristics between Spas.

	**Stufe di Nerone**	**Colli Asolani**	**Giardini Poseidon**
N. of total patients	70	30	60
Balneotherapy alone, n	30	30	60
Inhalation Therapy alone, n	36	-	-
Balneotherapy + Inhalation, n	4	-	-
N. of balneotherapy/daily	1	1	1
Session duration, minutes	20	20	20
Rest period, minutes	30	30	30
N. of inhalations/daily	1 direct jet + 1 aerosol	-	-
Session duration, minutes	10 min each		
Rest period, minutes	10 min between		
N. of Balneotherapy + Inhalation /daily	1 bath + 1 jet + 1 aerosol	-	-
Session duration, minutes	20 + 10 each		
Rest period, minutes	30 bath rest + 10 min between inhalations		
Total Spa Cycle Duration, days	12	12	12
Treatment Setting	Individual	Individual	Individual
Recruitment period	April 2023 – January 2024	April 2023 – January 2024	April 2023 – October 2024
Data Collection Period	April 2023 – January 2024	April 2023 – January 2024	April 2023 – October 2024

Supplementary Table S2. Comparisons between interventions.

	**Balneotherapy alone N = 57**					**Inhalation therapy alone N = 17**					
**Symptoms**	N	Before	After	Δ%	P value	N	Before	After	Δ%	P value	P value*
Chronic fatigue	43 (75)	3.2±0.8	1.6±1.0	-50%	0.001	4 (24)	3.3±0.5	3.0±0.0	-9%	0.39	<0.01
Muscle pain	33 (58)	3.2±0.9	1.7±1.1	-47%	0.001	2 (12)	3.0±0.0	1.5±2.1	-50%	0.5	0.01
Joint pain	27 (47)	3.0±0.9	1.7±1.0	-43%	0.001	4 (24)	3.5±0.6	1.3±1.5	-63%	0.12	0.08
Brain fog	21 (37)	2.8±1.1	1.5±0.9	-46%	0.001	3 (18)	3.3±0.6	3.0±0.0	-9%	0.42	0.14
Persistent cough	16 (28)	2.9±1.3	0.7±1.0	-76%	0.001	7 (41)	2.6±1.0	1.4±1.3	-46%	0.03	0.31
Headache	18 (32)	2.9±1.0	1.1±0.8	-62%	0.001	4 (24)	2.5±1.0	1.5±1.7	-40%	0.25	0.52
Taste disorders	9 (16)	2.6±1.0	0.7±1.1	-73%	0.003	3 (18)	3.0±0.0	2.0±1.0	-33%	0.23	0.86
Pseudo freezing	11 (19)	2.4±1.2	0.9±1.0	-62.5%	0.002	2 (12)	2.5±2.1	1.6±2.1	-36%	0.03	0.47
Tinnitus	10 (18)	2.7±0.9	1.9±1.0	-30%	0.04	3 (18)	3.7±0.6	1.7±1.2	-54%	0.07	0.99
Sore throat	9 (16)	2.7±1.1	0.3±0.7	-89%	0.001	1 (6)	4	0	-	-	NA
Chest pain	11 (19)	2.2±1.0	0.7±0.9	-68%	0.002	2 (12)	3.0±0.0	1.0±1.4	-67%	0.30	0.47
Persistent loss of smell	6 (11)	2.8±1.2	0.7±0.8	-75%	0.02	4 (24)	4.0±0.0	2.3±1.2	-43%	0.13	0.17
Dysphony	7 (12)	1.6±1.0	0.1±0.4	-94%	0.02	1 (6)	3	0	-	-	NA
Gastroesophageal reflux	10 (18)	2.8±0.9	1.1±0.7	-61%	0.001	1 (6)	3	0	-	-	NA
Nausea	7 (12)	3.0±0.6	0.4±0.5	-87%	0.001	0 (0)	-	-	-	-	NA
Itching	5 (9)	2.6±1.5	0.0±0.0	-100%	0.003	1 (6)	3	0	-	-	NA
Hair loss	8 (14)	2.4±1.3	1.0±0.5	-58%	0.02	1 (6)	3	2	-	-	NA
Burning skin sensation	8 (14)	2.9±0.6	0.8±0.9	-72%	0.001	1 (6)	3	3	-	-	NA
Nasal congestion	0 (0)					2 (12)	2.5±0.7	0.5±0.7	-80%	0.30	NA
Dysphagia	2 (4)	2.0±1.4	0.0±0.0	-100%	0.3	1 (6)	3	0	-	-	NA
Earache	3 (5)	2.7±0.6	1.3±0.6	-52%	0.06	2 (12)	3.0±0.0	2.5±0.7	-17%	0.5	0.35
Irritability	1 (2)	3	2	-	-	1 (6)	2	1	-	-	NA
Vomiting	3 (5)	3.3±0.6	0.3±0.6	-91%	0.04	0 (0)	-	-	-	-	NA
Dyspnea	9 (16)	2.6±1.4	0.7±0.8	-73%	0.006	2 (12)	2.0±0.0	1.5±0.7	-25%	0.5	0.68

*, Chi square test for Spa therapy effects (Δ% variations) between subjects who were treated with balneotherapy alone and inhalation therapy alone.

Supplementary Table S3. Quality of Life assessment before and after Spa therapy – Cohen d effect.

SF-36 items	**Coehn d effect Before Spa**	**Coehn d effect After Spa**
PF	-0.52	-0.18
RLP	-0.36	-0.19
P	-0.29	-0.39
GH	-0.18	-0.19
EF	-0.72	-0.55
RLE	-0.37	-0.22
SF	-0.39	-0.28
EWB	-0.70	-0.37

Abbreviations. PF, Physical Functioning; RLP, Role Limitations due to Physical health; RLE, Role Limitations due to Emotional problems; EF, Energy and Fatigue; EWB, Emotional Well-Being; SF, Social Functioning; P, Pain; GH, General Health perceptions.

Supplementary Table S4. Multiple logistic regression analysis in patients with long COVID-19.

Dependent variable = Spa therapy prescribed [Balneotherapy]	Estimate	SE	95%CI	P value
**Intercept**	-3.448	73.99	-149.0 to 155.3	0.9628
**Age (years)**	0.0143	0.04309	-0.07221 to 0.1037	0.7406
**Sex [Female]**	3.169	1.540	0.5013 to 6.757	0.0396
**Height (m)**	-3.422	43.99	-99.87 to 81.79	0.9380
**Weight (Kg)**	0.0583	0.5225	-0.9531 to 1.185	0.9111
**BMI**	0.2121	1.519	-2.894 to 3.277	0.8890
**PF before Spa**	-0.0304	0.03290	-0.1006 to 0.03201	0.3560
**PF after Spa**	-0.0253	0.04609	-0.1297 to 0.06026	0.5825
**RLP before Spa**	-0.0179	0.01470	-0.04976 to 0.009641	0.2211
**RLP after Spa**	-0.0658	0.03046	-0.1436 to -0.01701	0.0307
**P before Spa**	-0.0493	0.03285	-0.1272 to 0.008320	0.1332
**P after Spa**	0.0517	0.03713	-0.01373 to 0.1380	0.1639
**GH before Spa**	0.0383	0.05188	-0.06489 to 0.1491	0.4602
**GH after Spa**	0.0385	0.05197	-0.05650 to 0.1579	0.4584
**EF before Spa**	-0.0877	0.05309	-0.2067 to 0.006799	0.0985
**EF after Spa**	0.0979	0.07094	-0.03104 to 0.2567	0.1672
**SF before Spa**	0.0215	0.03526	-0.04691 to 0.09532	0.5423
**SF after Spa**	-0.0359	0.03793	-0.1163 to 0.03568	0.3436
**RLE before Spa**	0.0237	0.01354	-0.001096 to 0.05372	0.0806
**RLE after Spa**	0.0569	0.02801	0.008539 to 0.1205	0.0420
**EWB before Spa**	0.1231	0.06411	0.004881 to 0.2666	0.0549
**EWB after Spa**	-0.1395	0.08288	-0.3298 to 0.007381	0.0924
**Dependent variable = Sex [Female]**	**Estimate**	**SE**	**95%CI**	**P value**
**Intercept**	21.51	185.4	-391.7 to 403.4	0.9077
**Age (years)**	0.0346	0.07965	-0.1195 to 0.2287	0.6643
**Height (m)**	-12.15	113.2	-244.0 to 241.4	0.9145
**Weight (Kg)**	-0.9664	1.241	-4.641 to 1.061	0.4362
**BMI**	2.694	3.580	-3.167 to 13.33	0.4518
**Balneotherapy**	3.622	2.515	0.1592 to 10.17	0.1498
**PF before Spa**	-0.0464	0.05733	-0.1740 to 0.07997	0.4186
**PF after Spa**	-0.0266	0.1081	-0.3175 to 0.1578	0.8056
**RLP before Spa**	0.0013	0.03517	-0.07692 to 0.07399	0.9706
**RLP after Spa**	-0.0148	0.04874	-0.1097 to 0.09380	0.7622
**P before Spa**	-0.0494	0.05486	-0.2081 to 0.04576	0.3682
**P after Spa**	-0.0006	0.06712	-0.1420 to 0.1364	0.9930
**GH before Spa**	0.0427	0.07645	-0.1285 to 0.2267	0.5763
**GH after Spa**	0.0381	0.09926	-0.1645 to 0.3132	0.7008
**EF before Spa**	-0.2162	0.1058	-0.5018 to -0.04611	0.0410
**EF after Spa**	0.1181	0.1425	-0.1276 to 0.5049	0.4070
**SF before Spa**	0.1216	0.09718	-0.02082 to 0.3493	0.2107
**SF after Spa**	-0.2151	0.1021	-0.5236 to -0.06738	0.0350
**RLE before Spa**	0.0189	0.02460	-0.03466 to 0.07051	0.4417
**RLE after Spa**	0.01002	0.03543	-0.06645 to 0.08728	0.7773
**EWB before Spa**	0.1241	0.1391	-0.1027 to 0.5441	0.3724
**EWB after Spa**	0.0704	0.2252	-0.2939 to 0.5838	0.7546
**Dependent variable = anti-COVID-19 vaccination [YES]**	**Estimate**	**SE**	**95%CI**	**P value**
**Intercept**	-91.62	97.66	-304.1 to 116.2	0.3482
**Age (years)**	0.0316	0.05549	-0.07346 to 0.1611	0.5695
**Sex [Female]**	1.668	2.983	-3.784 to 8.951	0.5760

**Height (m)**	60.04	59.34	-65.24 to 189.2	0.3116
**Weight (Kg)**	-0.7768	0.7070	-2.335 to 0.6710	0.2719
**BMI**	2.533	2.085	-1.593 to 7.218	0.2244
**Balneotherapy**	-3.030	3.672	-12.11 to 2.361	0.4093
**N. of COVID-19 infections**	-0.5093	1.109	-3.040 to 1.703	0.6461
**PF before Spa**	0.0443	0.05295	-0.05722 to 0.1739	0.4027
**PF after Spa**	-0.0628	0.09468	-0.2750 to 0.09073	0.5073
**RLP before Spa**	0.0479	0.03253	-0.01211 to 0.1290	0.1404
**RLP after Spa**	0.0263	0.03201	-0.03629 to 0.09856	0.4114
**P before Spa**	-0.0826	0.05772	-0.2218 to 0.01036	0.1522
**P after Spa**	0.1022	0.06463	-0.004378 to 0.2554	0.1138
**GH before Spa**	-0.0166	0.06941	-0.1714 to 0.1161	0.8116
**GH after Spa**	-0.0657	0.07025	-0.2268 to 0.05601	0.3497
**EF before Spa**	-0.0289	0.07465	-0.2040 to 0.1202	0.6992
**EF after Spa**	-0.0145	0.08238	-0.1954 to 0.1564	0.8604
**SF before Spa**	0.0448	0.05101	-0.04844 to 0.1701	0.3793
**SF after Spa**	-0.01362	0.05203	-0.1264 to 0.08390	0.7934
**RLE before Spa**	0.0031	0.02382	-0.04815 to 0.05337	0.8979
**RLE after Spa**	-0.0538	0.04666	-0.1707 to 0.02226	0.2492
**EWB before Spa**	-0.0202	0.05442	-0.1429 to 0.08705	0.7112
**EWB after Spa**	-0.0991	0.08111	-0.3132 to 0.04105	0.2219

Abbreviations. SE, standard error; CI, confidential interval; PF, Physical Functioning; RLP, Role Limitations due to Physical health; RLE, Role Limitations due to Emotional problems; EF, Energy and Fatigue; EWB, Emotional Well-Being; SF, Social Functioning; P, Pain; GH, General Health perceptions.
